# COVID-19 Infection: A Sparked Ignition to a Thrombotic Storm

**DOI:** 10.7759/cureus.27038

**Published:** 2022-07-19

**Authors:** Yixin Zhang, Melissa Oye, Michael Omar

**Affiliations:** 1 Internal Medicine, University of Florida College of Medicine – Jacksonville, Jacksonville, USA; 2 Internal Medicine, University of Florida Health Jacksonville, Jacksonville, USA; 3 Internal Medicine and Cardiology, University of Florida College of Medicine, Jacksonville, USA

**Keywords:** hypercoagulable, arterial thrombosis, acute limb ischemia, covid-19, thrombotic storm

## Abstract

Thrombotic storm (TS) is a rare yet life-threatening condition that requires aggressive thrombolytic or anticoagulant therapy. Clinical manifestation of TS can be disastrous as it amplifies thrombotic pathways causing widespread organ ischemia. We present a patient who developed TS following a COVID-19 infection. He was simultaneously diagnosed with an ST-elevation myocardial infarction, multiple pulmonary emboli, aortic thrombi, and bilateral limb ischemia. Further workup was positive for a spindle cell neoplasm, which combined with the prothrombotic nature of COVID-19 infection likely produced an exaggerated response leading to a diffuse thrombotic event. Through this case, we would like to highlight the importance of having a collective field of expertise in making the most appropriate medical decision under critical situations.

## Introduction

The hallmark of a thrombotic storm (TS) is the concurrence of multiple thrombotic events affecting different vascular beds over a short time span [1]. It is a rare hematologic emergency that can be fatal if not managed promptly. Patients who develop TS often have an underlying hypercoagulable disorder followed by a secondary clinical insult (i.e., inflammation, infection, and trauma) that opens the flood gates to serial thrombosis [2]. Our current options for management include dual antiplatelet therapy, oral anticoagulation with vitamin K antagonist (i.e., warfarin) or direct oral anticoagulants (DOACs), and surgical or percutaneous vascular interventions. However, no clear guidelines exist on its management currently.

## Case presentation

A 53-year-old male with a past medical history of hypertension, coronary artery disease, and seizure disorder presented to the hospital with four days of progressive bilateral leg pain, left greater than right. Prior to the onset of leg pain, he endorsed one week of flu-like symptoms. On physical exam, he was tachycardic and hypertensive. His lower extremities were cool to touch (left worse than the right) with absent dorsalis pedis and popliteal pulses on the left and diminished distal pulses on the right. There were marked motor and sensory deficits in both legs.

**Figure 1:**
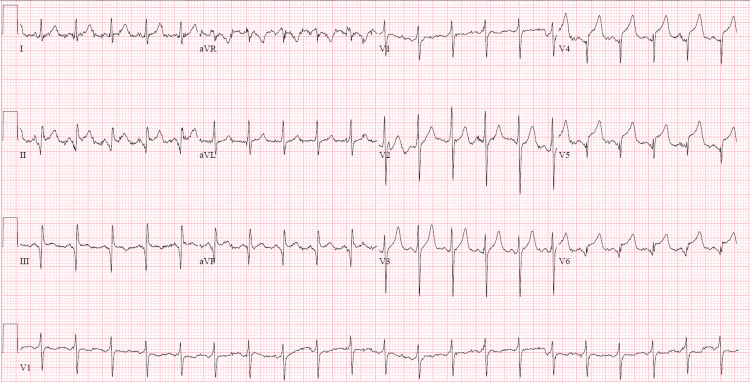
Initial ECG showing ST-segment elevations of the inferolateral leads

**Figure 2:**
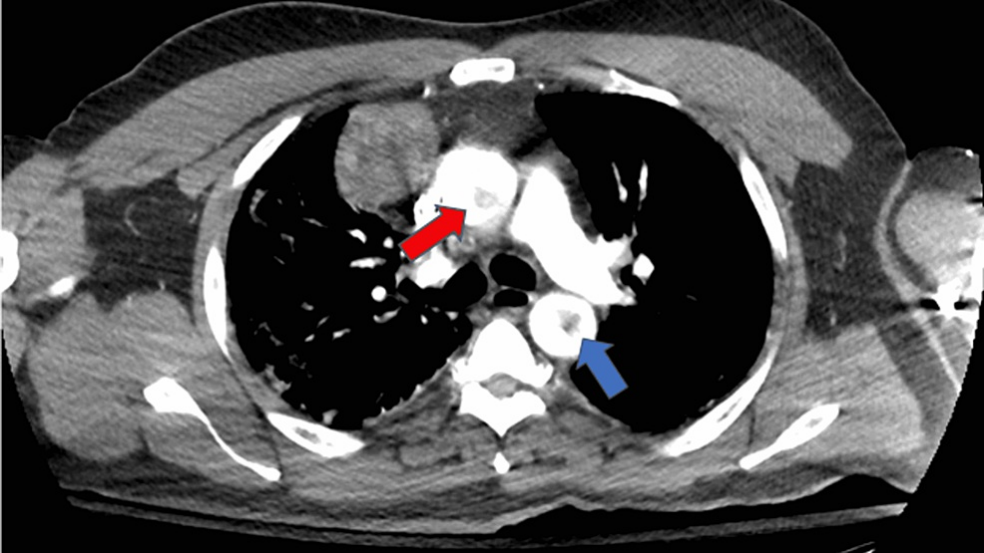
Thrombi in the ascending (red arrow) and descending aorta (blue arrow)

**Figure 3:**
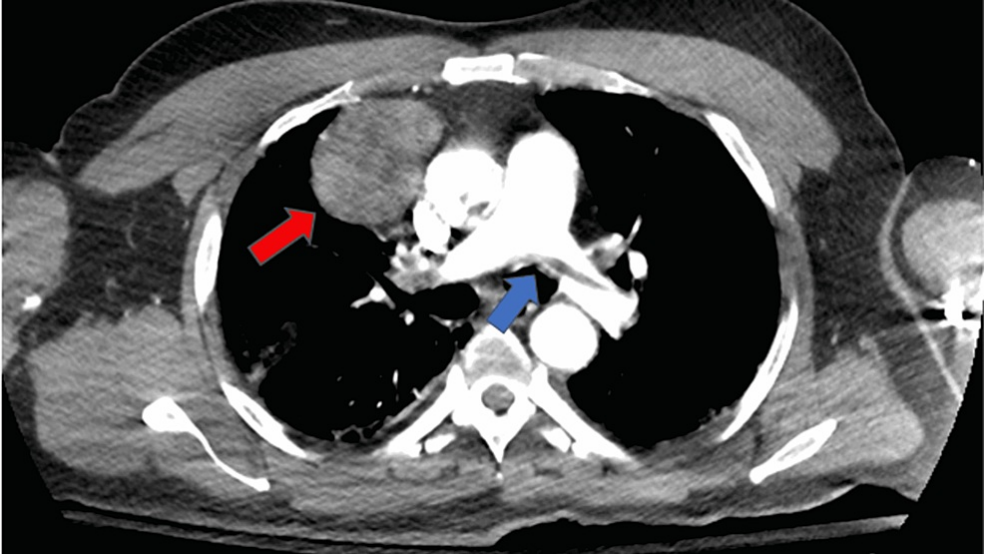
CT scan showing a right-sided anterior mediastinal mass measuring 6 cm x 4.8 cm x 7.1 cm (red arrow) and a thin saddle PE (blue arrow) PE: Pulmonary emboli.

**Figure 4:**
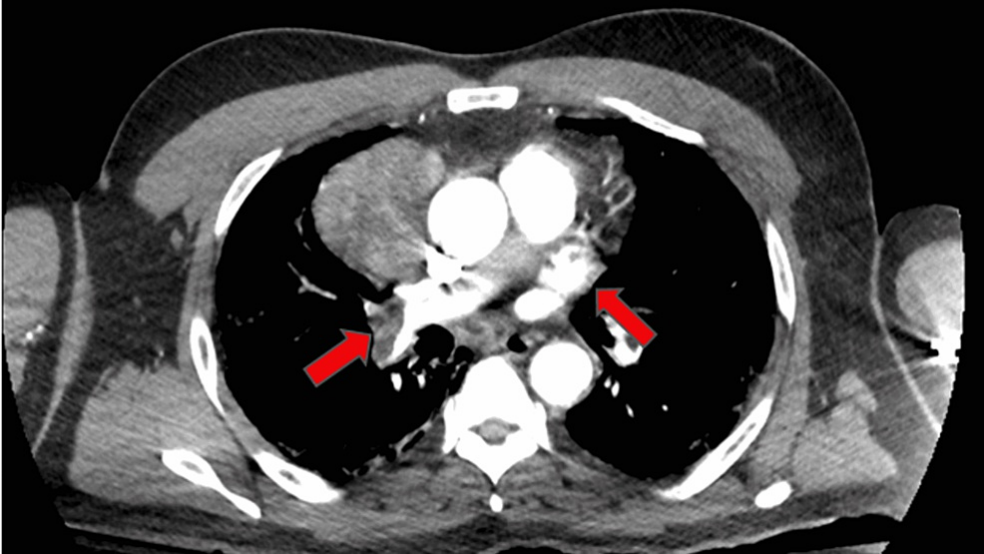
Bilateral PE of right and left main pulmonary arteries (red arrows) PE: Pulmonary emboli.

**Figure 5:**
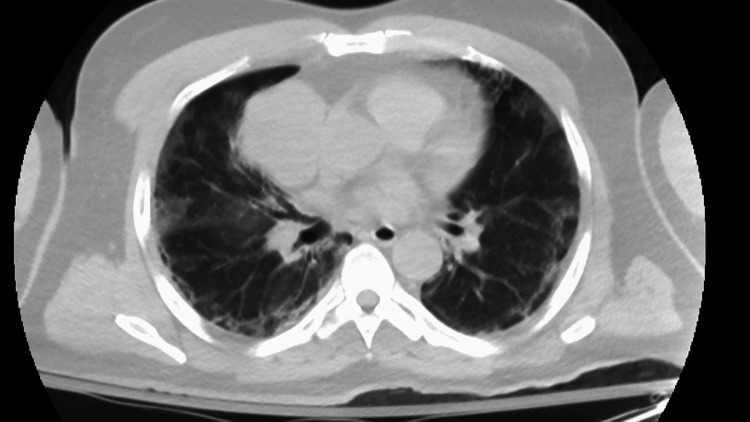
Extensive bilateral ground-glass opacities in the setting of COVID-19 infection

A complete metabolic panel was significant for acute kidney injury with a blood urea nitrogen (BUN) of 73 mmol/L, creatinine of 4.09 mmol/L, and hyperkalemia at 5.5 mmol/L. Complete blood count showed leukocytosis of 20,000/mm^3^ (reference: 4,000-11,000 mm^3^) with normal hemoglobin and platelet count. The high-sensitivity troponin assay was markedly elevated at 2,488 ng/L (reference < 22 ng/L). An initial electrocardiogram (ECG) revealed an ST-elevation myocardial infarction (STEMI) of the inferolateral leads, although the patient denied chest pain (Figure 1). A computed tomography angiogram (CTA) dissection protocol revealed bilateral pulmonary emboli (PE) with a thin saddle embolus, multiple intraluminal thrombi within the ascending and descending aorta, and abrupt occlusion of the left common femoral artery; there was also an incidental finding of a right anterior mediastinal mass measuring 6 cm x 4.8 cm x 7.1 cm (Figures 2-4). He was additionally found to be COVID-positive with CT findings of extensive pulmonary airspace disease (Figure 5).

Given the patient presented with three potentially life-threatening conditions, such as acute STEMI, acute limb ischemia, and bilateral central pulmonary emboli, an emergent multidisciplinary approach was essential. Percutaneous coronary intervention (PCI) was deferred by the interventional cardiology team as catheter access could potentially dislodge the aortic thrombi, leading to thromboembolic stroke. Given his acute STEMI, he was also a poor surgical option for thrombo-embolectomies and fasciotomies due to the high risk of major adverse cardiovascular events (MACE). Local catheter-directed thrombolytic therapies by the interventional radiology team would have been futile due to the extensive clot burden throughout his vasculature. Thus, despite the absence of hemodynamic instability, a collective decision was made to pursue systemic thrombolysis. Discussion of risks and benefits of each procedure and management was held with the patient. A decision was made, collectively with the patient, to proceed with systemic thrombolysis. Tenecteplase (TNK) was administered as a single bolus dose of 50 mg, followed by a heparin infusion, aspirin, and P2Y12 inhibitor loading (dual antiplatelet therapy). In this case, the P2Y12 inhibitor used was intravenous cangrelor as an off-label indication, given the patient's baseline anemia, high risk of bleeding, and the possible need for salvage surgical or percutaneous interventions.

Over the ensuing 12 hours, his repeat EKGs show resolution of his ST elevations. A repeat CTA dissection protocol showed resolved PEs, persistent aortic emboli, and unchanged complete occlusion of the left common femoral artery. Given the persistent aortic thrombi, he could not undergo left heart catheterization. He ultimately required bilateral above-the-knee amputations due to nonviable extremities. In regard to the mediastinal mass, a core biopsy revealed spindle cell neoplasm favoring thymoma. He is following up with oncology and cardiothoracic surgery for ongoing workup and treatment. Due to the severity of his presentation, he was placed on lifelong warfarin therapy with an international normalized ratio (INR) goal of 2.5-3.5. After a prolonged and complex hospital stay, he was discharged to a long-term care facility.

## Discussion

We presented a case of a critically ill patient with extensive small- and large-vessel arterial thrombi in the setting of underlying malignancy with an active COVID-19 infection. Both diseases pose a high risk for thrombotic events; thus, the combination of the two created a TS. The term "thrombotic storm" was first described by Kitchens et al. in 1998 when he summarized the key features after reporting six different cases of thrombotic events, as given in Table 1 [2].

**Table 1:** Five features of a thrombotic storm described by Kitchens et al.

Key features of a thrombotic storm
1. Underlying hypercoagulable disorder
2. Provocation to initiate thrombosis
3. Rapid development of new thrombosis
4. Response to prompt use of a thrombolytic agent or anticoagulant therapy
5. Remarkable good long-term prognosis if the cycle of thrombosis is interrupted

Based on reported cases, an acute clinical insult often precedes the start of a TS. For our patient, the underlying factors for a preexisting hypercoagulable state were the mediastinal spindle neoplasm. The COVID-19 infection acted as a spark to ignite an inextinguishable cytokine storm. Interestingly enough, about 50% of TS cases occur in individuals with known autoimmune conditions [2]. Other associated conditions are paroxysmal nocturnal hemoglobinuria, Crohn’s disease, polysubstance abuse, and pregnancy complications [3].

The treatment for TS in current reported literature is an aggressive anticoagulant or thrombolytic therapy for gravely ill patients followed by lifelong uninterrupted anticoagulation therapy [1-3]. We do not know the exact duration of thrombus presence in our patient but suspected a subacute time frame for the lower extremities. A collaborative treatment plan was made with a multidisciplinary team in which the treatment option would provide the most benefit and lowest mortality risk. As mentioned prior, our patient had an acute STEMI but was not a candidate for coronary catheterization due to the risk of thromboembolic stroke. The benefit of salvaging his lower limbs was greatly outweighed by the risks of cardiovascular adverse events, the risk of major bleeding while receiving medical management of STEMI with heparin and cangrelor, and the risk of death. Systemic thrombolysis was decided in an effort to restore blood flow to the multiple ischemic organs. Unfortunately, his limbs were non-salvageable after a delayed presentation on admission followed by postponed intervention.

This case summarized an unforgettable clinical vignette as we witnessed a merciless TS blazing into the large and small vessels of a patient with a preexisting hypercoagulable state. The excessive inflammation associated with COVID-19 infection can lead to thrombosis via platelet activation, endothelial cell activation/injury, and immune system activation [4]. Current guidelines from the American Society of Hematology (ASH) on the use of anticoagulation related to COVID-19 favor prophylactic over therapeutic doses in patients who do not have confirmed or suspected venous thromboembolism [5]. Our case raises an important question on whether patients who are at higher risk for venous thromboembolism should receive full-dose therapeutic anticoagulation at the time of diagnosis as a preventive treatment for TS.

## Conclusions

Lastly, the TS remains a topic with a myriad of potential for advanced medical and clinical research since there is no clear consensus or guidelines on its treatment. Its presentation often requires immediate medical and invasive management due to the life-threatening conditions caused by multiple thrombotic events. Unfortunately, patients who survive the attack may be left with permanent health damages. Our patient will need rigorous physical rehab and lifelong uninterrupted warfarin therapy since this is the only way to avoid recurrent thrombosis.
